# Altered brainstem responses to modafinil in schizophrenia: implications for adjunctive treatment of cognition

**DOI:** 10.1038/s41398-018-0104-z

**Published:** 2018-03-06

**Authors:** Michael J. Minzenberg, Jong H. Yoon, Steffan K. Soosman, Cameron S. Carter

**Affiliations:** 1 Department of Psychiatry and Biobehavioral Sciences, University of California, Los Angeles, CA 90024 USA; 2 Department of Psychiatry and Behavioral Sciences, Stanford School of Medicine, and the VA Palo Alto Health Care System, Palo Alto, CA 94304 USA; 3 Department of Psychiatry, University of California, Davis School of Medicine, Sacramento, CA 95816 USA; 4 Program in Neuroscience, University of California, Davis, CA 95817 USA

## Abstract

Candidate pro-cognitive drugs for schizophrenia targeting several neurochemical systems have consistently failed to demonstrate robust efficacy. It remains untested whether concurrent antipsychotic medications exert pharmacodynamic interactions that mitigate pro-cognitive action in patients. We used functional MRI (fMRI) in a randomized, double-blind, placebo-controlled within-subject crossover test of single-dose modafinil effects in 27 medicated schizophrenia patients, interrogating brainstem regions where catecholamine systems arise to innervate the cortex, to link cellular and systems-level models of cognitive control. Modafinil effects were evaluated both within this patient group and compared to a healthy subject group. Modafinil modulated activity in the locus coeruleus (LC) and ventral tegmental area (VTA) in the patient group. However, compared to the healthy comparison group, these effects were altered as a function of task demands: the control-independent drug effect on deactivation was relatively attenuated (shallower) in the LC and exaggerated (deeper) in the VTA; in contrast, again compared to the comparison group, the control-related drug effects on positive activation were attenuated in LC, VTA and the cortical cognitive control network. These altered effects in the LC and VTA were significantly and specifically associated with the degree of antagonism of alpha-2 adrenergic and dopamine-2 receptors, respectively, by concurrently prescribed antipsychotics. These sources of evidence suggest interacting effects on catecholamine neurons of chronic antipsychotic treatment, which respectively increase and decrease sustained neuronal activity in LC and VTA. This is the first direct evidence in a clinical population to suggest that antipsychotic medications alter catecholamine neuronal activity to mitigate pro-cognitive drug action on cortical circuits.

## Introduction

Schizophrenia is a common, severe, high-impact disorder with cognitive deficits representing a critical determinant of clinical outcome^1,2^. Cognitive impairment is particularly important among processes that are highly dependent on cortical networks operated by the prefrontal cortex (PFC)^3,4^. Presently, there is no established treatment for cognitive impairment in schizophrenia, and therefore, advances in this research area have the potential to alleviate a considerable global illness burden.

Two major candidate neural targets for the remediation of PFC dysfunction in schizophrenia are the catecholamine systems arising from the pontine locus coeruleus (LC) and midbrain ventral tegmental area (VTA), which use norepinephrine (NE) and dopamine (DA) respectively as neurotransmitters. These systems project widely throughout the cortex, and are well-suited to modulate widely-distributed neural networks such as those engaged by the PFC during higher-order cognition. Indeed, there is ample evidence in both animal models and humans that NE and DA strongly modulate PFC neurons, PFC-operated networks, and PFC-dependent cognitive processes such as working memory and cognitive control^5–9^.

**Fig. 1:**
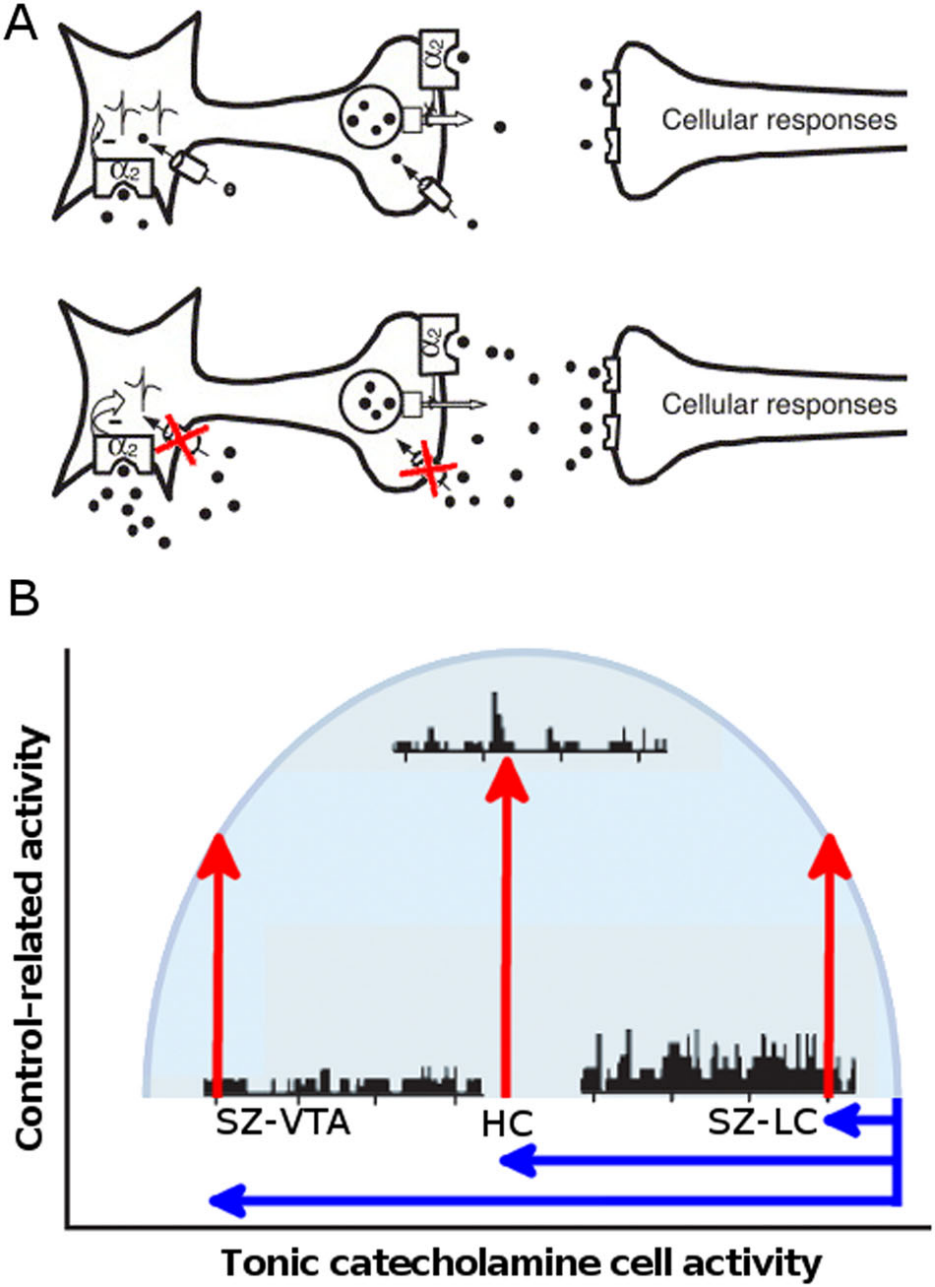
**a** Cellular model of modafinil action on LC-NE neuron, indicating how NET inhibition (red Xs on schematic neuron in second row) by modafinil increases NE at the cell-body α_2_ autoreceptor, leading to moderately-slowed tonic discharge rate, and increased NE release (via both enhanced task-related discharge and terminal NET inhibition). **b** Hypothetical model of inverted-U curve relating control-related catecholamine cell activity to tonic activity, and the relative effects of NET inhibition by modafinil in healthy subjects (HC) vs. subjects with schizophrenia (SZ). The present results suggest that compared to HC, the SZ group exhibits relatively less control-independent deactivation in the LC (blue), relatively greater control-independent deactivation in the VTA (blue), and less control-related positive activation in both LC and VTA (red). Figures modified from Invernizzi and Garattini, 2004, and Aston-Jones and Cohen, 2005

Models of catecholamine neuronal activity suggest that optimization of cortical networks and cognition arises from robust catecholamine neuronal responses to task-relevant information. These responses in turn require a moderate level of tonic, background activity, which tends to be a function of behavioral state^5,10^ (see inverted-U-shaped curve in Fig. 1b). Computational modeling suggests that modest slowing of tonic background activity of catecholamine neurons may be ideal for enhancing their responsiveness to task-relevant stimuli^11^. This can be achieved pharmacologically with moderate inhibition of plasma-membrane catecholamine transport, which increases catecholamine-mediated activation of inhibitory cell-body autoreceptors^12,13^ (Fig. 1a). We have previously shown that modafinil, a low-potency inhibitor of transporters for NE (NET) and DA (DAT)^14,15^ (reviewed in ref. 16) modulates the LC and the cortical network subserving cognitive control, in a manner consistent with this cellular model of pro-cognitive action^17^. This mechanism therefore serves as an important model for the pharmacological modulation of PFC-based networks to remediate cognition in schizophrenia, and could form the basis for modafinil effects on brain function and cognition observed in other studies of schizophrenia^18^.

Despite the intense interest and resources that the scientific, clinical, regulatory communities and industry have dedicated to cognition in schizophrenia, to date numerous pharmacological strategies (targeting a wide range of neurochemical systems) have shown minimal or inconsistent efficacy in clinical trials^19–24^. One important and under-addressed factor driving these results could be that antipsychotic medications mitigate the impact of candidate pro-cognitive agents through pharmacodynamic mechanisms. For example, antipsychotics exert strong effects that decrease neuronal activity in VTA-DA neurons^25^ while increasing tonic activity in LC-NE neurons^26–30^. These effects would then alter the responses of these brain areas to candidate agents that modulate catecholamine systems. Interactions with concurrent antipsychotics could occur regardless of the neurochemical system targeted by a given pro-cognitive agent, due to the highly interacting nature of neurotransmitter systems in the brain, where biogenic amines mutually influence each other as well as amino acid neurotransmission^31,32^.

We therefore adopted an experimental medicine approach, using fMRI to support a mechanistic, model-driven investigation of drug action on modulation of large-scale neural systems that support complex cognition. We tested modafinil modulatory effects on the LC, VTA and cortical networks during cognitive performance in schizophrenia, integrating both cellular and systems-level neurobiological models. We report novel evidence that the catecholamine modulatory effects of modafinil are observable in schizophrenia in support of cognition, yet critically, these effects are altered in a pattern consistent with the actions of concurrent antipsychotic medications.

## Subjects and methods

### Subjects/overview of procedures

The study was set in the University of California—Davis Medical Center from February 2007 to July 2010. The ClinicalTrials.gov identifier is NCT00423943. All procedures were approved by the UCD Institutional Review Board. All subjects provided informed consent for all procedures, and received remuneration for procedures, and the study was concluded at the end of the funding award period. There were no changes to the methods or outcomes after trial commencement. Subjects were all outpatients, recruited from the community and our research clinic at UCD, and were included if they were aged 18–50 years, and lacked the following history: neurological illness, including head injury with loss of consciousness, uncorrectable visual or peripheral motor disturbance; full-scale IQ < 70 (by Wechsler Abbreviated Scale of Intelligence: WASI); known intolerance to modafinil; active substance abuse within 6 months of study; uncontrolled medical illness; incompatability with MRI procedures. All patients were evaluated by structured diagnostic interview, using the SCID-I with DSM-IV-TR criteria, and all assigned a 295.X diagnosis. Each subject completed a double-blind, placebo-controlled crossover study of modafinil (single oral dose 200 mg) effects on neural activity measured by fMRI during cognitive control task performance. BOLD signal change measured by fMRI during the cognitive task was the primary outcome measure, and task performance was the secondary outcome measure. To address concurrent medication effects in relation to altered modafinil effects on BOLD signal change, we also derived measures of medication load at catecholamine autoreceptor subtypes, conferred by the schizophrenia patients’ treatment regimens, as determined by published standard indices of in vitro catecholamine receptor activity (see Supplement). We tested these hypothesized relationships by correlating concurrent medication loads with neural responses to modafinil, as an additional secondary outcome measure. Tertiary outcome measures included subjective state measured with the POMS, and vital signs measured with a blood pressure cuff. While this was an unprecedented, preliminary study, sample size was approximated based on both preliminary results from the healthy comparison group^17^ and an earlier published study of modafinil effects on prefrontal cortical function^33^.

See Supplemental Methods for additional detail on the evaluation of subjects.

### Cognitive Paradigm

The Preparing to Overcome Prepotency (POP) Task^17^ (Supplemental Fig. 1) was presented using EPrime software. In this task, a visual cue (Red or Green color patches, 500 ms duration) in the center of the visual field instructs a stimulus-response (S–R) mapping to the probe, which occurs after a delay (7.5 s delay from cue offset to probe onset). The probe is an arrow that occurs with equal frequency pointing to the left or right, presented in the center of the visual field for 500 ms in duration. The direction of the arrow probe is randomized to preclude the preparation of specific motor responses in the cue-probe delay period. The response demand is prepotent for Green-cued trials (i.e., the correct response is a left button-press for leftward arrows, and right for right), and is non-prepotent for Red-cued trials (i.e., left button-press for rightward arrows, and vice versa). The period from probe onset to cue onset of the successive trial is 12 s. During both cue-probe delay and probe-cue interval, subjects are instructed to fixate visually on a crosshair presented in the center of the visual field. Four blocks with 20 trials each were performed, with randomized order of cues, 70% of which were comprised of prepotent (Green-cued) S–R mappings, and each block lasting 6 min 40 s. Subjects were instructed to ‘go as fast as you can without making mistakes.’

The POP task requires cognitive control to overcome a prepotent stimulus-response mapping. High-control demands (Red-cued trials) are associated with decrements (costs) in response accuracy and speed, and robust activity of the lateral and medial PFC during the preparatory cue-probe delay period^16^.

### fMRI Acquisition and Pre-processing

Event-related fMRI was conducted on a 3 tesla Siemens Trio MRI system with a Siemens 8-channel phased array coil. Measurement of Blood Oxygen Level-Dependent (BOLD) contrast was conducted during single-shot, echo-planar imaging (EPI), using a T2*-weighted sequence, and whole-brain coverage. The parameters of the EPI sequence were TR 2000 ms, TE 30 ms, flip angle 90°, FOV 220 × 220 mm, with 36 contiguous slices in the axial oblique plane with voxel size 3.4 mm isotropic. In addition, a structural MRI was acquired for normalization of EPI images to the template, using a Magnetization-Prepared Rapid Acquired Gradient Echo (MP-RAGE) sequence, with the following parameters: TR 2500 ms, TE 4.82 ms, acquisition time 9:20, flip angle 7°, FOV 256 × 256 mm, with 192 slices 1 mm thick. Pre-processing and analysis of EPI images were performed using SPM5. The first four images (preceding onset of trial one of block one) were discarded to allow for stabilization of the scanner signal. The remaining images were realigned (motion-corrected) to the first retained image in the first block, and adjusted for acquisition time (slice timing correction). At this point, skull-stripping was performed on both MP-RAGE images and the single-subject T1 template from SPM. The MP-RAGE image from each subject was co-registered to the T1 template to determine normalization parameters, which were then applied to each EPI image for that subject via 6-parameter rigid-body affine transformation to standard MNI space. Images were then resliced to 2 × 2 × 2 and spatially smoothed with an 8 mm, full-width-at-half-maximum Gaussian kernel. At this point, Drug and Placebo day scans were concatenated for modeling of the signal and inferential testing.

#### Modeling and inferential testing of voxel-wise drug effects on neural activity

Derivation of the signal proceeded with the use of the General Linear Model. Regressors were established for Drug_RedCues, Drug_GreenCues, Placebo_RedCues, and Placebo_GreenCues, and analogous regressors for Probes. We also included a nuisance regressor for errors and trials lacking a motor response, to account for these event-related signal changes, but did not include these in the inferential testing, as they were too rare for reliable analysis (see task performance results). A canonical (double-Gaussian) hemodynamic response function was convolved with a series of delta functions to model the BOLD time series, with regressors placed at cue and probe onset. We also established the temporal derivative of the HRF as a regressor, paired with each of the experimental condition regressors, to account for temporal variation in the latency of the event-related response. A 0.0125 Hz high-pass filter was used, a first-order autoregressive function to account for serial autocorrelations, and grand mean scaling to account for global differences in signal value across test days. After signal estimation, linear contrasts were defined at the single-subject level (see below), and then relevant contrast maps from individual subjects (containing voxel-wise parameter estimates for a given contrast) were entered into group-level analysis for inferential testing.

The control-independent Treatment effect was tested with the contrast defined as (Drug_RedCue + Drug_GreenCue) > (Placebo_RedCue + Placebo_GreenCue). This contrast uses all available data to estimate treatment effects that are independent of Task Condition, i.e., cognitive control demands. We hypothesized that in this contrast, significant drug effects (relative to placebo) would be manifest as deactivations in both the LC and the VTA, attendant to the model of catecholamine neurotransmission at cell bodies, an effect well-established for catecholamine transporter inhibitors such as stimulants and antidepressants^34^. In contrast, for the control-related treatment effect on brain activity, the contrast was established as (Drug_RedCue minus Drug_GreenCue) > (Placebo_RedCue minus Placebo_GreenCue). This is analogous to a directional test of the Treatment-by-Task Condition interaction in ANOVA terminology, and in this contrast, we hypothesized that the Drug would serve to positively enhance LC and VTA activity. This is because despite a control-independent effect of Drug manifest as deactivation, the drug should augment the positive difference in activity related to cognitive control demands (i.e., Red Cue over Green Cue-related activity), as the cellular model suggests that a modest decrease in tonic firing rates should be associated with an increased phasic (i.e., task-related) firing rate, as an expression of gain control in neural activity^5^. This contrast also tested the hypothesis that elsewhere in the cognitive control network, the drug would be associated with a significantly greater enhancement of positive control-related BOLD signal change, again compared to placebo. All contrast maps are depicted at a threshold of *p* < 0.05, with clusters corrected to *p* < 0.05 by small-volume (for LC and VTA) or the False Discovery Rate (for the cortical cognitive control network).

### Code availability

All neuroimaging data pre-processing and analyses were conducted using SPM5 software, which is readily available in the public domain.

See the Supplemental Methods for details of localization of the LC, VTA and cognitive control network in EPI images, and correlation analysis of BOLD signal change with antipsychotic receptor-binding affinities.

## Results

See Supplemental Results for task performance results

### fMRI results within schizophrenia group

**Fig. 2:**
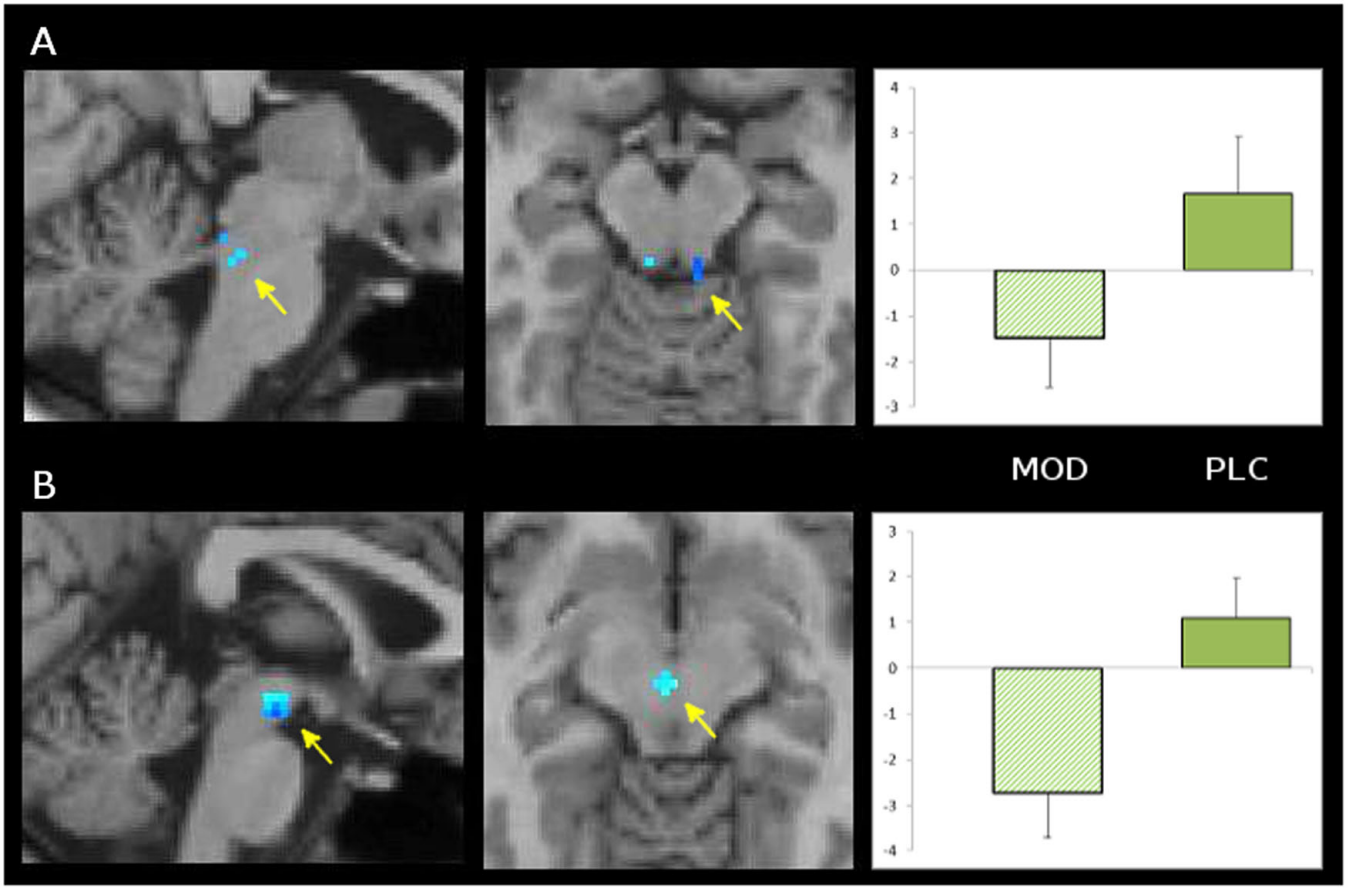
Modafinil effects on control-independent activity in locus coeruleus and ventral tegmental area in Schizophrenia Group. Contrast maps of SZ group for Control-independent effect of Modafinil Treatment on decreased activity in locus coeruleus (**a**) and ventral tegmental area (**b**). Clusters depicted are small-volume-corrected to *p* < 0.05. Bar graph depicts mean betas (±s.d.) in statistically significant voxels for MOD (left) and PLC (right). See text for details of data acquisition and analysis. See Supplemental Table 5 for characteristics of clusters

The SZ group showed a significant control-independent effect of Treatment (i.e., across both cue types) in both the locus coeruleus (Fig. 2a; Supplemental Table 5) and ventral tegmental area (Fig. 2b; Supplemental Table 5). No control-independent Treatment effects meeting the FDR-corrected significance level were observed elsewhere in the brain (data not shown). Control-related Treatment effects (i.e., on the Red Cue minus Green Cue difference) were also interrogated both within the LC and VTA, as well as throughout the rest of the brain. Here, no effects were observed that met the corrected significance level.

### fMRI results comparing schizophrenia group to healthy control group

**Fig. 3:**
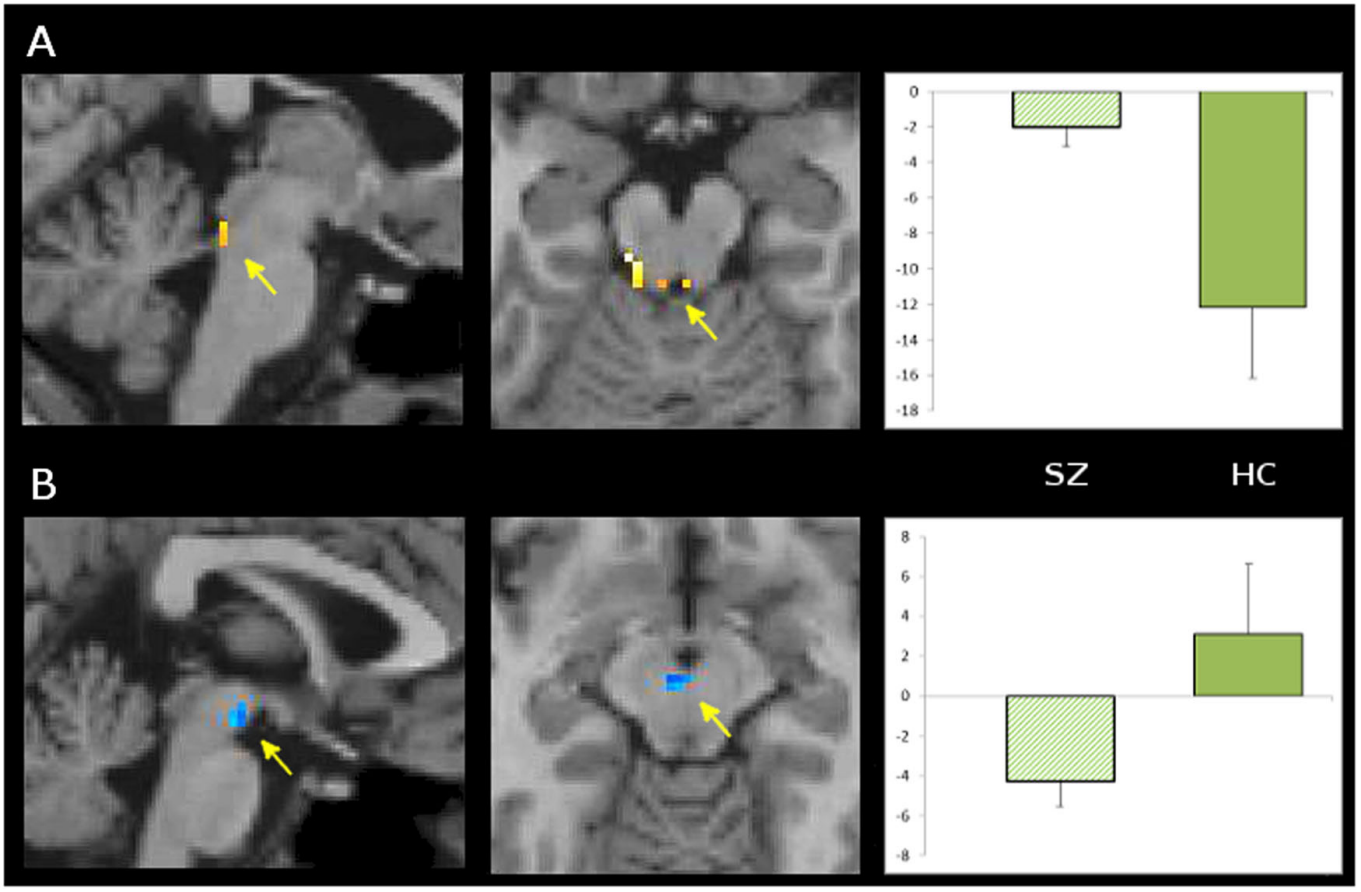
Modafinil effects on control-independent deactivation in schizophrenia group compared to healthy control group are shallower in locus coeruleus and deeper in ventral tegmental area. Contrast maps of SZ group minus HC group, for Control-independent effect of Modafinil Treatment, on activity in locus coeruleus (**a**) and ventral tegmental area (**b**). Clusters depicted are small-volume-corrected to *p* < 0.05. Bar graph depicts mean betas (±s.d.) in statistically significant voxels for SZ (left) and HC (right) groups. Note that the SZ group, compared to the HC group, shows relatively greater activity in LC, i.e., less deactivation, and relatively less activity in VTA, i.e., greater deactivation. See Supplemental Table 5 for characteristics of clusters

**Fig. 4:**
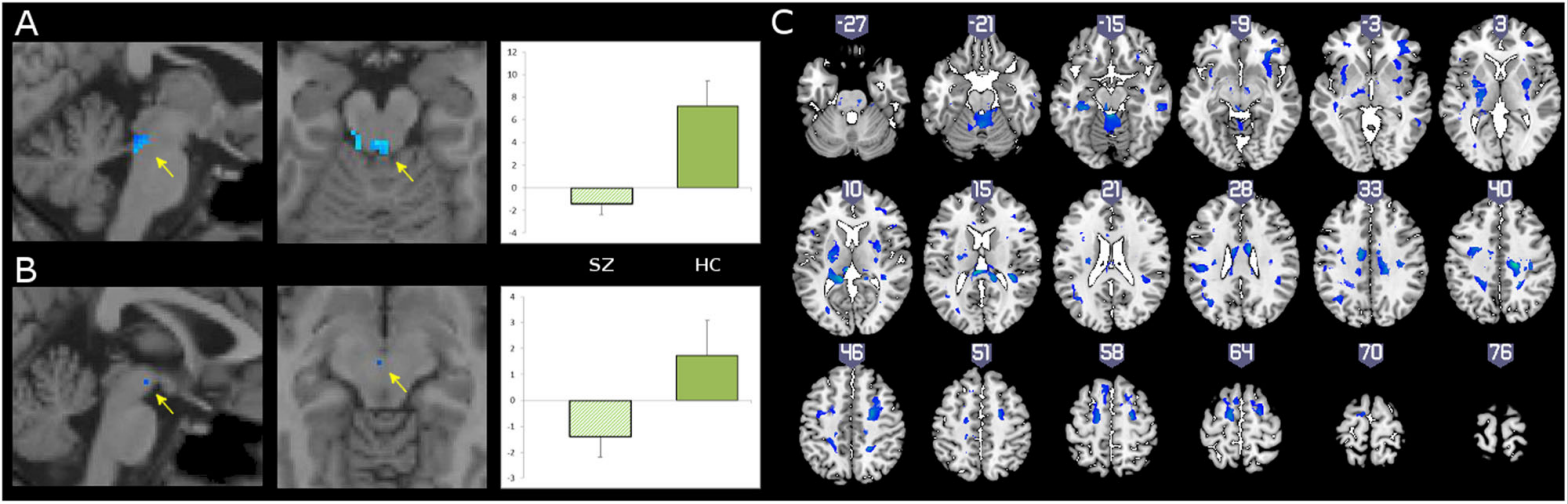
Modafinil effects on control-related positive activation in locus coeruleus, ventral tegmental area and neocortical cognitive control network are impaired in schizophrenia group compared to healthy control group. Contrast maps of SZ group minus HC group, for Control-related effect of Modafinil Treatment on activity within locus coeruleus (**a**), ventral tegmental area (**b**), and brain as a whole (**c**). Bar graph depicts mean betas (±s.d.) in statistically significant voxels for SZ (left) and HC (right) groups. Note that the SZ group, compared to the HC group, shows relatively impaired Treatment effects on control-related activity in both subcortical catecholamine brain regions as well as in various regions of the fronto-subcortical cognitive control network. See Supplemental Table 5 for characteristics of clusters meeting corrected threshold

Compared to the HC group, the SZ group showed altered control-independent Treatment effects, with relatively shallower (i.e., attenuated) deactivation in the LC (Fig. 3a; Supplemental Table 5), and deeper (i.e., stronger) deactivation in the VTA (Fig. 3b; Supplemental Table 5). For the control-related treatment effect, the SZ group was impaired relative to the HC group in both the LC and VTA (Fig. 4a,b; Supplemental Table 5). In the cognitive control network, the control-related treatment effect of modafinil was impaired in the SZ group in several regions (Fig. 4c, Supplemental Table 5). These included PFC regions in the bilateral superior/medial frontal gyrus (primarily in supplementary motor area and pre-SMA), right inferior frontal gyrus (in ventrolateral PFC), and bilateral middle frontal gyrus (in premotor cortex); and bilateral middle and posterior cingulate gyrus. More posterior regions included temporoparietal areas such as the right middle temporal gyrus, and bilateral inferior parietal lobule. Subcortical areas included the cerebellar vermis, left hippocampus, and bilateral putamen extending into thalamus. No regions were observed that exhibited a stronger control-related Treatment effect in the SZ group compared to the HC group.

### Association of catecholamine receptor actions of concurrent medications with modafinil effects in LC and VTA

We evaluated how the patients’ concurrent antipsychotic medications may relate to altered brainstem responses to modafinil, as a function of antagonism of catecholamine receptors that serve as autoreceptors (Supplemental Methods).

#### LC effects

**Fig. 5:**
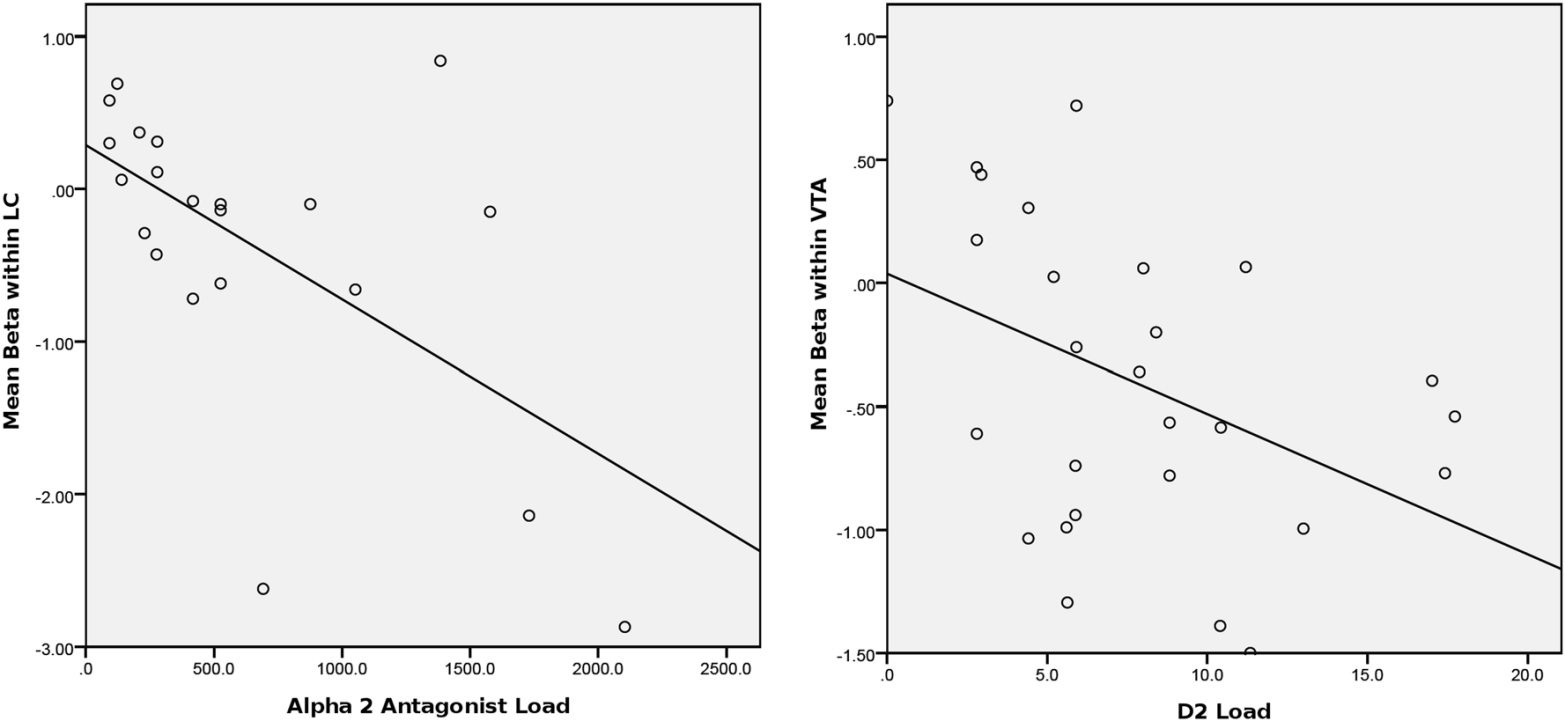
Relationship of modafinil effects in LC and VTA to catecholamine receptor antagonism by concurrent antipsychotic medications. **a** Control-related effect of modafinil in LC is attenuated by α_2_ antagonism of concurrent antipsychotic medications. Scatterplot of mean betas in control-related modafinil effect in LC, as a function of α_2_ receptor loads (in haloperidol equivalents) conferred by patients’ antipsychotic medications. Subgroup (*n* = 21) on monotherapy with atypical antipsychotics. *r* = −0.60, *p* = 0.005. **b** Control-Independent effect of modafinil on deactivation in VTA is positively related to D2 antagonism by concurrent antipsychotic medications. Scatterplot of mean betas in VTA in response to control-independent modafinil effect (Drug minus Placebo), as a function of D2 receptor loads conferred by patients’ antipsychotic medications (*n* = 27). *r* = −0.42, *p* = 0.031

The α_2_ receptor load of the patients’ concurrent atypical antipsychotic medications was reasonably normally distributed across the sample (skew = 1.24, kurtosis = 0.62), as was the control-related modafinil effect on LC activity (skew = −0.92, kurtosis = 0.65). This α_2_ receptor load was strongly inversely related to control-related modafinil effects on LC activity (*r* = −.60, *p* = 0.005; Fig. 5a). These correlations persisted upon controlling for D_2_ load (*r*_partial_ = −.57, *p* = 0.009) and muscarinic load (*r*_partial_ = −.58, *p* = 0.007), suggesting a specific relationship between α_2_ receptor load and LC effects that was not accounted for by other neurochemical actions nor the total dose of the antipsychotic medications. Furthermore, muscarinic load was not associated with control-related modafinil effects on LC activity (*r* = .16; *p* = .50). These results indicate that α_2_ receptor antagonism of the patients’ concurrent antipsychotic medications was strongly and specifically related to impaired modafinil effects on control-related LC activity.

#### VTA effects

The D_2_ receptor load of the patients’ antipsychotic medications was normally distributed across the sample (skew = 0.76, kurtosis = 0.13), as was the VTA response to modafinil (skew = 0.18, kurtosis = −0.83). This D_2_ load was significantly related to modafinil effects on VTA deactivation (*r* = .42, *p* = 0.031; Fig. 5b). There was a modest decrement in this association when controlling for global SAPS scores (*r*_partial_ = .33, *p* = 0.10). Muscarinic load was not associated with modafinil effects on VTA deactivation (*r* = −.13; *p* = 0.52). These results indicate that D_2_ receptor antagonism of the patients’ concurrent medications was moderately and specifically related to stronger deactivating effects of modafinil in the VTA.

## Discussion

In this study, we provide the first available evidence that fMRI can be used to demonstrate the modulatory effects of a drug with pro-cognitive potential on activity in the locus coeruleus and the ventral tegmental area, in a cognitively-impaired clinical population during cognitive performance. We found that modafinil administration leads to relative deactivation in LC and VTA in schizophrenia patients, consistent with predictions of control-independent effects arising from NET and DAT inhibition at cell bodies in these subcortical regions (Fig. 1a). Importantly, these effects were altered compared to a healthy control group, with two lines of evidence suggesting an interaction with the concurrent antipsychotic medications prescribed to these patients was responsible for these altered responses. First, relative to the healthy control group, the patients responded to modafinil with shallower deactivation in LC and deeper deactivation in VTA. This pattern is consistent with the underlying effects of chronic antipsychotic medication treatment, which lead to sustained decreases in tonic firing in the VTA, and increases in tonic firing in the LC. Second, these altered modafinil effects in the LC and VTA were significantly correlated respectively with antagonism at α_2_ and D_2_ receptors (despite the relatively simple measure of receptor load used here), These receptor subtypes serve an important regulatory autoreceptor-mediated inhibition of NE and DA neurons, respectively. In the patients overall, the consequences of altered responses to modafinil was that the drug was less able to positively modulate control-related increases in activity, in both these subcortical regions and in neocortical and subcortical terminal fields that support cognitive control (see Fig. 1b for hypothetical model of altered modafinil effects on catecholamine neuron activity in SZ). These findings have significant potential implications for the prospects of candidate drugs to normalize cortical dysfunction in schizophrenia, to remediate impaired cognition.

Chronic treatment with either typical or atypical antipsychotics leads to sustained increases in firing of LC neurons^26–30^. The tonic disinhibition of LC-NE neurons may then mitigate or override the feedback inhibition of LC-NE activity via cell-body autoreceptors, which would be manifest as the shallower control-independent deactivation in patients that we observed by fMRI. Given the relationship of tonic to phasic LC activity^5^, proxied here respectively as control-independent and control-related BOLD signal change, this increased tonic LC activity (unnormalized by modafinil) would impede relative control-related increases in LC-NE activity. We have observed just this combination of control-independent and control-related drug effects in our patient sample. It remains unclear which monoamine receptor(s) mediates the antipsychotic effect on LC activity. However, we found that the α_2_ antagonist load of the concurrent antipsychotics was strongly and specifically related to impaired modafinil effects on control-related LC activity. This convergent evidence suggests that antagonism of the cell-body α_2_ autoreceptor leads to impaired optimization of control-related phasic LC activity. This evidence supports the model we outlined previously, where modafinil action to inhibit NET at LC cell bodies leads to autoreceptor-mediated slowing of control-independent LC activity, allowing optimized control-related phasic LC activity to effectively modulate the cognitive control network (Fig. 1b)^17^.

In contrast to the activating effects on the LC, chronic antipsychotic treatment induces depolarization inactivation in VTA-DA neurons, rendering the cell-body less able to generate action potentials (reviewed in^25^). The relatively depolarized (but not discharging) state of antipsychotic-exposed VTA-DA neurons may then render these neurons relatively more sensitive to DAT inhibitor effects that increase DA at cell bodies. This increased sensitivity could also result from relatively more D_2_ receptors in a high-affinity state, which is induced with chronic antipsychotic treatment and associated with the D_2_ affinity of these medications^35^. One of the present findings, that the deactivating effect of modafinil on the VTA was significantly related to the D_2_ load conferred by the patients’ concurrent antipsychotic medications, suggests that D_2_-receptor effects interact with modafinil responses in the VTA. Exaggerated deactivation of the VTA to modafinil could render these neurons suboptimally-responsive to control-related excitatory inputs, leading to the dissociation of cell-body vs. terminal effects of DAT inhibition (see penultimate paragraph below). This effect may also interact with underlying pathophysiology in this system, manifest in either neurochemical disturbances and/or altered responses to cognitive demands^35–39^. These considerations highlight the utility of an experimental medicine approach using *in vivo* methods such as fMRI, to afford direct tests in patient populations of model-driven predictions about the pharmacological modulation of these systems during cognitive processes^40^.

The emphasis here on cell-body effects of modafinil, and modulation of neuronal activity in the LC and VTA, is entirely compatible with models of catecholamine function that emphasize post-synaptic actions in the PFC^4,6,9^. Phasic activity in both VTA^10^ and LC neurons^41,42^ leads to greater neurotransmitter release compared to tonic activity, and optimizes throughput in active cortical ensembles, which may be mediated by D_1_^6,7^ or α_2_^9^ receptors. It remains likely that modafinil exerts important actions at NET in terminals in the PFC, probably to amplify the beneficial effects of cell-body modulation. The D_2_-mediated antipsychotic effects observed here could also relate to actions at DA terminals in the PFC, and α_2_ antagonist effects also manifest directly at post-synaptic receptors in PFC, both leading to altered descending cortical input to the LC and VTA. Antipsychotic treatment may in fact induce a state where cell-body, terminal and post-synaptic actions are uncoupled in response to catecholamine transport inhibition. In this scenario, antipsychotic treatment leads to: (1) altered effects of pro-cognitive NET/DAT inhibition on cell-body activity (observed here), combined with (2) increased release of residual NE and DA, resulting from both terminal autoreceptor antagonism (by antipsychotics) plus NET and DAT inhibition (by modafinil), but in a manner uncoupled to cell-body activity (and the influence of control-related excitatory input to catecholamine neurons from the PFC and elsewhere), and (3) direct antagonism by antipsychotics of post-synaptic D_2_ and α_2_ receptors in the cortex. Thus, the circuit that maintains bidirectional influence of these subcortical neurochemical systems with cortical networks would be disrupted, unable to respond to a modulatory drug with pro-cognitive potential such as modafinil.

This study is limited by a rather modest sample size, and that the antipsychotic medication treatment was naturalistic and not randomized nor blinded, unlike the single dose of modafinil. Nevertheless, the correlations of BOLD signal change in response to modafinil exhibited specificity in their relationships with monoamine receptor-mediated effects of antipsychotics, which were predicted based on the known neurochemical effects of both modafinil and antipsychotics, and were generally not attributable to overall illness severity as measured by psychotic symptoms nor proxied by other neurochemical effects of the antipsychotics. In addition, many of these subjects were concurrently prescribed other psychotropic agents (and in a few cases, non-psychiatric medications, for other medical conditions). It remains unclear if these other medications may have contributed to the altered neural responses to modafinil observed here. Nonetheless, the pattern of observed effects fit very well the predicted pattern based on the actions of antipsychotic drugs on these systems, and how they would be expected to interact with modafinil. In addition, the supplemental medications are reasonably-representative of the adjunctive treatments that are routinely used with schizophrenia outpatients, suggesting that the observed altered brain responses are likely representative of those that may be found among schizophrenia patients more generally.

In addition, the patient sample was quite characteristic of outpatient, community-dwelling populations with schizophrenia, in terms of demographics, symptomatology and functional status. These observations suggest that the present findings may have general relevance for the future clinical management of outpatients with schizophrenia, and the potential challenge of resolving treatment for psychotic symptoms that are the clinical hallmark of the disorder, with the cognitive impairment, which is not a defining feature of schizophrenia yet remains a major determinant of functional outcome.

It is very important to emphasize that in no way do these considerations suggest that modern antipsychotic medications be abandoned or that their use be curtailed. These medications are the mainstay of treatment of all psychotic disorders, and remain the most important advance in the history of schizophrenia treatment^43^. It is nonetheless interesting to consider how an antipsychotic agent with relatively less catecholamine antagonist activity may mitigate these problems and serve as a better alternative, in combination with novel pro-cognitive agents, to facilitate the development of new treatments for cognition in this illness.

## Electronic supplementary material

Supplement

Supplemental Figure 1

Supplemental Figure 2

Supplemental Figure 3
