# Supplementary material for: Altered brainstem responses to modafinil in schizophrenia: implications for adjunctive treatment of cognition
Source: Transl Psychiatry. 2018 Mar 6;8:58. doi: 10.1038/s41398-018-0104-z (PMC5838154; doi:10.1038/s41398-018-0104-z)
Supplement: Supplementary file 1 — Supplement [file 41398_2018_104_MOESM1_ESM.doc]

**Supplemental Methods and Results**

Subject Evaluation and Procedural Details

Diagnosticians were doctoral-level trained clinicians who underwent formal SCID training and achieved criterion for reliability. In addition, all diagnoses were confirmed by consensus case conference involving all diagnosticians. Three patients were assigned a diagnosis of schizoaffective disorder, depressive type, and all other patients a diagnosis of schizophrenia. All patients were clinically-stable, with no hospitalizations or changes in medication regimen for at least two months prior to study (see Supplemental tables 2 and 3 for demographics, symptom measures and medication regimens for all included patients). All subjects were instructed to maintain their usual quantities and patterns of nicotine and caffeine intake, without changes on or between test days, to avoid neural/cognitive effects due to changes in intake from their baseline. All included subjects tested negative for drugs of abuse in the urine on each testing day. Concurrent medications prescribed for non-psychiatric medical problems were rare in this sample: one subject each was taking omeprazole and atorvastatin, and two subjects were taking oral contraceptives. Randomization of the within-subjects cross-over treatment order was performed without stratification, with a computer algorithm by a research pharmacist, who also packaged active medication (which was purchased from the manufacturer at market price) and placebo in identical-appearing capsules for administration, and was otherwise uninvolved in the study.

15 patients completed the active drug testing day first, and 12 completed the placebo day first. In the patient subgroup off-ceiling for performance, 11 performed the drug day first, and 8 performed the placebo day first. The mean test day interval was 9.9 ± 7.2 days (minimum 3 days), which ensured full washout of the drug between test days. All subjects received the dose of either active drug or placebo in the morning with the measurement immediately thereafter of baseline blood pressure and heart rate, and completion of the baseline Profile of Mood States self-report questionnaire (POMS-State version). Immediately prior to MRI scanning, blood pressure and heart rate measurement was repeated; echo-planar imaging was initiated at an average elapsed time 3.7 ± 0.3 hours post-dose, within the time window of reported group-averaged peak plasma levels of modafinil ^S1^. After scanning, all subjects completed the POMS again. No subjects reported any adverse subjective or physical symptoms during study procedures. All investigators remained blind to treatment order for individual subjects until all data was acquired for that subject and treatment order information was then necessary to sort neuroimaging data for inferential testing.

Eleven SZ patients in total consented for the study and were randomized but then failed to provide data for inclusion in the analysis: 4 consented and then withdrew without further contact, prior to any study procedures; 1 had repeated positive urine drug test results and was excluded from further participation; 2 experienced aborted MRI procedures due to intolerance of the procedure (1 for chest discomfort and 1 for anxiety/paranoia); 3 had excessive movement in the scanner (each with ≥ 10 mm translation in one plane, with numerous transient shifts); and 1 exhibited floor performance (random accuracy on Red Cue trials on placebo, and with excessive trials without a motor response, suggesting that the patient was not engaged in the task). The latter four subjects were excluded by investigators blinded to their fMRI results. The remaining 27 patients each completed all study procedures without significant adverse effects; each of these subjects exhibited less than one voxel of movement in each translational plane in all MRI sessions, and data for this sample was fully-analyzed. See Supplemental Table 1 flow diagram of study enrollment as per CONSORT guidelines ^S2^ (Moher *et al*, 2001).

The healthy comparison (HC) subjects were all recruited from the community, and were included if they were free of past or present psychiatric illness as determined by the Structured Clinical Interview for DSM-IV Disorders, Non-Patient version; lacked both significant medical problems and a family history of major psychiatric illness by report; and were not taking medications with either psychotropic effects or established drug-drug interactions with modafinil at study. The modafinil effects in the HC group (who were enrolled in the same experimental protocol as the patient sample) have been reported previously ^16^. In the present report, the HC group results are utilized solely for between-group comparisons with the SZ group (See Supplemental Table 2 for demographic characteristics of each group).

Localization of LC and VTA in BOLD images

Given our hypotheses directed at Treatment effects in the LC and VTA, we defined small volumes for interrogation of both LC and VTA. We maintained a voxel-wise statistical analysis, rather than data reduction in a region-of-interest analysis, to permit the evaluation of topographic heterogeneity within these subcortical structures. There is evidence for heterogeneity in the source of projections to the frontal cortex, versus subcortical targets for instance, arising from the rodent LC ^S3^ (Berridge and Waterhouse, 2003). It remains unknown if this variation of ascending projections exists in the human LC; heterogeneous response properties in subregions within the LC would be of interest in characterizing the functional anatomy of LC modulation. As described below, for both the LC and VTA, we utilized unbiased, functionally-defined topographic regions for which localization was confirmed by reference to easily-discriminable adjacent spatial landmarks and/or structurally-defined masks in the template brain.

For the LC, this consisted of voxels in standard MNI space that were A) located in the rostrodorsal pons, near the 4th ventricle/sylvian aqueduct, and restricted to the pons (e.g., not extending rostrally into the mesencephalon or superiorly into the 4th ventricle) by an anatomically-defined pontine mask drawn on the T1 template (using MRIcro) by the investigators previously to this study, in a manner blind to the fMRI results ^16^; and B) which showed significant association of BOLD signal change with pupil diameter, measured by pupillometry during POP Task performance in an independent sample of nine healthy adults without medication treatment (and not members of the HC comparison group), whose imaging data was otherwise acquired and processed in a manner identical to the present sample (without a drug intervention). The pupil time-series of each of these nine subjects was used as a parametric regressor in the model of the BOLD time-series, and pupil-defined voxels in the pons (60 voxels, equal to 480 mm^3^) were identified in the contrast versus an implicit baseline (see Supplemental Figure 2). Pupil diameter closely tracks LC single-unit activity ^5^ and therefore provides an objective, functionally-defined strategy for LC localization.

For the VTA, we generated a sphere with radius 3 mm (approximately one voxel in a single dimension), centered on the ventral midbrain maximum found in the reward paradigm reported in ^S4^ (Knutson *et al*, 2005). To our knowledge, this paper by Knutson and colleagues reports on the most widely-adopted reward paradigm used for fMRI to interrogate the role of the VTA-ventral striatal system in reward processing. This cluster of voxels was centered on the midline of the mesencephalic neuraxis, rostral to the pontomesencephalic flexure and caudal to the mammillary bodies, superior to the interpeduncular fossa, and ventral/ventromedial to both the substantia nigra and red nucleus, and it shared no voxels with either of these bilateral structures (nor the subthalamic nucleus) as they are delineated in the PickAtlas library of topographic masks ^S5^ (Maldjian *et al*, 2003).

This strategy for both LC and VTA localization thus integrates functionally- and anatomically-defined criteria to reliably localize these structures. It also avoids the limitations associated with the use of specialized structural MRI images (such as neuromelanin-sensitive MRI), which are likely to considerably underestimate the volume and topographic extent of these nuclei. For these voxel-wise tests, the threshold for statistical significance was set at p < .05, with a small-volume correction using the LC and VTA (thus defined) separately as small-volumes.

Interrogation of treatment effects in the cognitive control network

The same statistical contrast for the control-related Treatment effect (defined above) was utilized to test hypothesized drug effects in the cognitive control network, within the SZ group. We created a mask to interrogate only voxels that showed an effect of cognitive control demand, defined as voxels with significant activity in either (Placebo_RedCue minus Placebo_GreenCue) or (Drug_RedCue minus Drug_GreenCue), each at p<.05 uncorrected. We then tested the control-related treatment effect in the SZ group with the contrast (Drug_RedCue minus Drug_GreenCue) > (Placebo_RedCue minus Placebo_GreenCue), with FDR correction to p < 0. 05.

To interrogate the control-related Treatment effect in the cognitive control network between groups, we created a mask with voxels that showed a control-related drug effect in either group, defined as voxels with significant activity in the contrast (Drug_RedCue minus Drug_GreenCue) > (Placebo_RedCue minus Placebo_GreenCue), within either diagnostic group at a voxel-wise threshold of p < 0.05 uncorrected. We then interrogated voxels within this mask as an unbiased test of hypothesized drug effects between groups, with FDR correction to p < 0.05. For this between-group test, we used the contrast [HC (Drug_RedCue minus Drug_GreenCue) > (Placebo_RedCue minus Placebo_GreenCue)] minus [SZ (Drug_RedCue minus Drug_GreenCue) > (Placebo_RedCue minus Placebo_GreenCue)]. This effectively tests the Group-by-Treatment-by-Task Condition interaction, with a test that permits directional inferences about a differential control-related Treatment effect between Groups. We hypothesized that A) the control-related Treatment effect would be manifest in the cognitive control network in the patient group, but that B) this effect in the patient group would be impaired relative to the control group.

Evaluation of the Relationship Between Antipsychotic Receptor-Mediated Effects and BOLD Response to Modafinil in LC and VTA

To address concurrent medication effects in relation to altered modafinil effects on BOLD signal change, we derived measures of load at catecholamine autoreceptor subtypes, conferred by the schizophrenia patients’ concurrent treatment regimens, as determined by published standard indices of *in vitro* catecholamine receptor activity. Load is defined here as the product of each patient’s total daily dose and the *in vitro* activity at a given receptor subtype, relative to a standard medication (see below). This measure provides a reasonably-straightforward method to evaluate relationships between receptor-mediated effects of prescribed medications and neuroimaging measures.

To evaluate α_2_ antagonist effects of antipsychotics on LC responses (as a test of the cellular model of pro-cognitive LC action), we first identified the subgroup of patients who were receiving atypical antipsychotic monotherapy (n=21). We emphasized this drug class because atypicals exhibit greater α_2_ antagonism than typical antipsychotics, this newer class is now widely considered the first-line treatment for schizophrenia, and considerations of cognitive effects of antipsychotics have been primarily concerned with this class. The α_2_ antagonist load for each of these patients was computed relative to haloperidol, using a published index that comprehensively integrates the *in vitro* α_2_ antagonist literature on antipsychotics ^S6^. We then conducted zero-order bivariate correlations of α2 antagonist load with the mean beta values within the LC (defined above), representing the modafinil effect on control-related LC activity; and repeated these as partial correlations, controlling for (in parallel analyses) the load at D2 and muscarinic receptors (defined below), to test whether α_2_ antagonist effects were confounded by these other neurotransmitter effects.

Analogously, we evaluated D2 antagonist effects on VTA responses by computing D2 load for all concurrent antipsychotics in all patients, according to a recently-published standard index for D2 antagonism that accounts for all atypical antipsychotics prescribed to the present patient sample, with D2 load relative to haloperidol ^S7^. To test whether antipsychotic actions at D2 autoreceptors related to altered BOLD response to modafinil in the VTA, D2 load was correlated with the modafinil effect on VTA deactivation (mean beta values within VTA, defined above). Finally, we computed anticholinergic load (relative to benztropine) for all concurrent medications for each patient (including intrinsic anticholinergic activity of antipsychotics such as olanzapine, and non-antipsychotic medications), also using a published standard index ^S8^. This accounted for another possible source of medication-related effects on neural/cognitive activity. Muscarinic load was correlated with both LC and VTA beta values separately as a test of specificity of the hypothesized autoreceptor-neuroimaging associations.

**Supplemental Results**

Demographic and Clinical Measures

The SZ group was significantly younger and less-educated than the HC group, with a trend toward lower intelligence (Supplemental Table 2). None of the other demographic measures were significantly different between the diagnostic groups. We addressed the group difference in age by conducting bivariate correlations between age and beta values from every experimental fMRI measure (i.e., control-independent and control-related Treatment effects, each in LC and VTA). Here, we found no significant correlations between age and any fMRI measure, either within each diagnostic group separately or across diagnostic groups (all Pearson r values between –.16 and +.15; all p values > 0.45). The only exception to this pattern was a significant negative correlation between age and the control-related Treatment effect in LC, within the SZ group (r = -.40, p = 0.04). Because the SZ group had both younger age and less control-related Treatment effect compared to the HC group, the age difference between groups would tend to offset the group difference in Treatment effect, rather than account for this difference. In sum, there was no evidence that the group difference in age was related to any group differences in fMRI measures.

The SZ group showed no significant effects of Treatment (Modafinil compared to Placebo) on peripheral hemodynamic parameters (all t < 0.53, all p > 0.6 by paired t test of pre- to post-treatment change scores) (Supplemental Table 4). Drug treatment (Modafinil compared to Placebo) was associated with modest, trend-level subjective effects to decrease Fatigue (t = -1.51, p = 0.072) and increase Vigor (t = 1.70, p = 0.050) scores (both one-tailed paired t tests of pre- to post-treatment change scores), and no other effects on subjective state measures by the POMS (Supplemental Table 4).

Performance in Full Schizophrenia Group

The full patient group mean accuracy and reaction time (RT) in each Treatment and Task Condition are shown in Supplemental Figure 3. In ANOVA of task accuracy in the full group of schizophrenia patients, there were significant main effects of Treatment (F = 4.94, df = 1,26; p = 0.035) and Task Condition (F = 39.76, df = 1,26; p < 0.0005); the interaction of Treatment and Task Condition was not significant (F = 2.23, df = 1,26, p = 0.15). ANOVA of RT showed significant main effect of Task Condition (F= 20.74, df 1,26, p < 0.0005), but no effect of Treatment (F = 2.47, p = 0.13) nor the Treatment-by-Task Condition interaction (F = 0.09, p = 0.77).

Performance in Schizophrenia Subgroup with Off-Ceiling Performance

As can be inferred from Supplemental Figure 3, a large share of patients performed at ceiling accuracy, rendering modafinil treatment effects more difficult to discern. We therefore identified a SZ subgroup (N=19) who performed off-ceiling on placebo, using the criterion of < 95% accuracy in the high-control (red-cue) condition on placebo. In this subgroup, ANOVA of task accuracy showed significant main effects of Treatment (F = 10.92, df =1,18, p = 0.004), Task Condition (F = 52.19, df =1,18, p < 0.0005) and the Treatment-by-Task Condition interaction (F = 5.53, df = 1,18, p = 0.030). ANOVA of RT showed a significant main effect of Task Condition (F = 11.30, df 1,18, p = 0.003), but no effects of Treatment (F = 2.50, p = 0.13) nor the Treatment-by-Task Condition interaction (F = 0.23, p = 0.64). To determine the direction of the Treatment-by-Task Condition interaction we conducted a paired t test. This showed that modafinil Treatment was associated with significantly reduced (i.e. improved) accuracy cost (Drug accuracy cost versus Placebo accuracy cost: t = -2.35, df = 26, p = 0.030).

Performance Across Both Diagnostic Groups

The within-HC group performance analysis has been previously reported ^16^. In ANOVA of task accuracy across both groups, there were significant main effects of Diagnostic Group (F= 10.93, df = 1, p = 0.002), Treatment (F= 5.79, df = 1,46; p = 0.020), Task Condition (F= 49.69, df = 1,46; p < 0.0005), and the Diagnostic Group-by-Task Condition interaction (F= 6.19, df = 1,46; p = 0.017). The Treatment-by-Task Condition interaction showed a trend-level effect (F= 3.07, df = 1,46; p = 0.086). The other interaction terms were non-significant (all F < 1.0; all p > 0.35). In ANOVA of RT, there was a significant main effect of Task Condition (F= 52.90, df = 1,46; p < 0.0005); all other main effects and interaction terms were non-significant (all F < 1.9; all p > 0.18). Because there were no significant effects of any interaction term that includes both Diagnostic Group and Treatment factors, we conclude that there were no differential Treatment effects on task performance between the two diagnostic groups, and have omitted follow-up T tests for brevity.

Supplemental References

S1 Robertson P, Hellriegel ET. Clinical pharmacokinetic profile of modafinil. *Clinical pharmacokinetics* 2003; **42(2):** 123-137.

S2 Moher D, Schulz KF, Altman D. The CONSORT statement: revised recommendations for improving the quality of reports of parallel-group randomized trials. JAMA 2001; **285(15):** 1987-1991.

S3 Berridge CW, Waterhouse BD. The locus coeruleus-noradrenergic system: modulation of behavioral state and state-dependent cognitive processes. *Brain research Brain research reviews* 2003; **42(1):** 33-84.

S4 Knutson B, Taylor J, Kaufman M, Peterson R, Glover G. Distributed neural representation of expected value. *The Journal of neuroscience: the official journal of the Society for Neuroscience* 2005; **5(19):** 4806-4812.

S5 Maldjian JA, Laurienti PJ, Kraft RA, Burdette JH. An automated method for neuroanatomic and cytoarchitectonic atlas-based interrogation of fMRI data sets. *NeuroImage* 2003; **19(3):** 1233-1239.

S6 Minzenberg MJ, Yoon JH. An index of relative central alpha-adrenergic receptor antagonism by antipsychotic medications. *Experimental and clinical psychopharmacology* 2011; **19(1):** 31-39.

S7 Andreasen NC, Pressler M, Nopoulos P, Miller D, Ho BC. Antipsychotic dose equivalents and dose-years: a standardized method for comparing exposure to different drugs. *Biological psychiatry* 2010; **67(3):** 255-262.

S8 Minzenberg MJ, Poole JH, Benton C, Vinogradov S. Association of anticholinergic load with impairment of complex attention and memory in schizophrenia. *The American journal of psychiatry* 2004; **161(1):** 116-124.

**Supplemental Figure 1. Diagram of POP Task structure.**

**Supplemental Figure 2. Location of Pupil-Related Locus Coeruleus in Standard-Space.**

Group-level contrast depicting cluster in rostro-dorsal pons containing voxels with a significant association of pupil diameter with BOLD signal fluctuation, in independent sample (n=9) of healthy subjects during POP Task performance. This cluster served as the pupil-defined locus coeruleus mask to interrogate modafinil treatment effects in SZ.

**Supplemental Figure 3. Modafinil Effects on Performance in Schizophrenia Group.**

Group means ± Standard Deviation.

**Supplemental table 1. CONSORT Flow Diagram.**

**Allocation**

**Analysis**

**Follow-Up**

**Enrollment**

Assessed for eligibility (n= 87)

Randomized (n=38)

Excluded (n= 49 total)

♦  Not meeting inclusion criteria (n= 29)

♦  Declined to participate (n= 20)

Analysed (n= 27)
♦ Excluded from analysis (n=4 total) (n=3 excessive movement in MRI; n=1 floor [random] task performance)

Lost to follow-up during/after intervention (n=0)

Discontinued intervention (n= 2 total) Intolerance of MRI (n=1 chest discomfort; n=1 anxiety/paranoia)

Allocated to intervention (n= 38)

♦ Received allocated intervention (n=33)

♦ Did not receive allocated intervention (n=5 total) n=4 lost to follow-up prior to intervention; n= 1 excluded after consent due to repeated positive urine drug tests

**Supplemental Table 2. Demographic and Clinical Characteristics of Schizophrenia Patients and Healthy Control Subjects.**

| **Measure** | **Schizophrenia Group (n=27)** |  | **Healthy Control Group (n=21)** |  |
| --- | --- | --- | --- | --- |
| Male | 19 (70%) |  | 12 (57%) |  |
| Right-Handed | 26 (96%) |  | 19 (90%) |  |
|  | **Mean** | **SD** | **Mean** | **SD** |
| Age | 26.2 ** | 8 | 33.3 | 8.2 |
| Parental Education | 15.1 | 1.9 | 14.5 | 3.0 |
| Subject Education | 13.1 *** | 1.6 | 16.0 | 2.4 |
| Full-Scale IQ (WASI) | 103 (*) | 13 | 110 | 11 |
| **Clinical Measures** | | | | |
| GAF | 49 | 11 | N/A |  |
| **SANS (mean global)** | | | | |
| Aff Flat | 1.8 | 1.2 | N/A |  |
| Alogia | 1.4 | 1.1 | N/A |  |
| Avolition | 2.3 | 1.3 | N/A |  |
| Anhedonia | 2.9 | 1.4 | N/A |  |
| Attention | 1.6 | 1.3 | N/A |  |
| **SAPS (mean global)** | | | | |
| Hallucinations | 1.1 | 1.5 | N/A |  |
| Delusions | 1.1 | 1.1 | N/A |  |
| Bizarre Behav | 0.1 | 0.4 | N/A |  |
| Form Thought | 0.4 | 0.8 | N/A |  |

(*) p<.10; ** p<.005; *** p<.0005 by two-tailed t test.

**Supplemental Table 3.**

**Concurrent psychotropic medications prescribed to schizophrenia patients at study.**

| Subject | Antipsychotics (daily dose, mg) | Other Medications (daily dose, mg) |
| --- | --- | --- |
| SZ 01 | Olanzapine 20 | Fluoxetine 80; Diphenhydramine 50 |
| SZ 02 | Risperidone 8 | Buproprion 100 |
| SZ 03 | Fluphenazine 5.4 | Benztropine 2 |
| SZ 04 | Aripiprazole 10 | None |
| SZ 05 | Aripiprazole 20 | None |
| SZ 08 | Aripiprazole 15 | None |
| SZ 12 | Quetiapine 1000 | None |
| SZ 13 | Quetiapine 800 | None |
| SZ 14 | Thiothixene 30 | Clonazepam 1; Venlafaxine 150; Trazodone 200; Varenicline 2 |
| SZ 15 | Haloperidol 8 | None |
| SZ 17 | Olanzapine 15 | Benztropine 4; Venlafaxine 225; Topiramate 200 |
| SZ 18 | Quetiapine 400 | Sertraline 200 |
| SZ 20 | Fluphenzine 10 | Paroxetine 40 |
| SZ 22 | Risperidone 2 | Benztropine 0.5 |
| SZ 23 | Aripiprazole 20 | Benztropine 1; Citalopram 20; Lamotrigine 100 |
| SZ 25 | Risperidone 2 | None |
| SZ 26 | None | None |
| SZ 27 | Olanzapine 15 | None |
| SZ 28 | Aripiprazole 15 | None |
| SZ 29 | Risperidone 2 | None |
| SZ 30 | Quetiapine 400;  Olanzapine 30 | Gabapentin 100 |
| SZ 32 | Ziprasidone 160  Thiothixene 30 | None |
| SZ 34 | Aripiprazole 30 | None |
| SZ 35 | Olanzapine 45 | None |
| SZ 36 | Risperidone 4 | Benztropine 1; Fluoxetine 20; Valproic Acid 1500 |
| SZ 37 | Aripiprazole 30 | Lorazepam 4 |
| SZ 38 | Risperidone 6 | None |

**Supplemental Table 4. Changes in Peripheral Hemodynamic Measures and Self-Reported Subjective Mood State (From Pre- to Post-Dose) After Modafinil Versus Placebo, in Schizophrenia Group. (*) p<.10 by one-tailed paired t test.**

|  | **Modafinil** |  | **Placebo** |  |
| --- | --- | --- | --- | --- |
| **Measure** | **Mean** | **SD** | **Mean** | **SD** |
| **Vital Signs** | | | | |
| Systolic Blood Pressure | 2.2 | 14.3 | 0.3 | 10.0 |
| Diastolic Blood Pressure | 5.4 | 10.7 | 4.5 | 8.6 |
| Heart Rate | -5.7 | 13.9 | -6.4 | 10.0 |
| **Profile of Mood States** | | | | |
| Tension | -0.09 | 3.13 | -0.39 | 2.93 |
| Depression | -0.44 | 2.27 | -0.70 | 2.01 |
| Anger | 0.48 | 2.45 | -0.48 | 2.57 |
| Fatigue | -0.83 (*) | 4.14 | 0.57 | 3.38 |
| Confusion | 0.35 | 2.01 | -0.30 | 1.15 |
| Vigor | 1.39 (*) | 4.44 | -5.83 | 19.87 |

**Supplemental Table 5. Modafinil Effects on Brain Activity during Cognitive Control in SZ.**

| **Contrast** | **Brain Region** | **BA** | **Cluster Size (mm^3^)** | **Peak T statistic** | **Peak MNI Coordinates** |
| --- | --- | --- | --- | --- | --- |
| **SZ Group** | | | | | |
| **Control-independent Drug Effect on Deactivation** | | | | | |
| Left Locus Coeruleus | | | 8 | -3.93 | -4, -32, -14 |
| Right Locus Coeruleus | | | 104 | -3.44 | 8, -34, -14 |
| Ventral Tegmental Area | | | 152 | -3.44 | -2, -18, -14 |
| **SZ Group versus HC group** | | | | | |
| **Control-independent Drug Effect: Shallower Deactivation in SZ Group** | | | | | |
| Left Locus Coeruleus | | | 176 | +4.27 | -10, -26, -16 |
| Right Locus Coeruleus | | | 64 | +3.36 | 8, -34, -12 |
| **Control-independent Drug Effect: Deeper Deactivation in SZ Group** | | | | | |
| Ventral Tegmental Area | | | 72 | -2.24 | -2, -16, -12 |
| **Control-related Drug Effect: Impaired Activation in SZ Group** | | | | | |
| Left Locus Coeruleus | | | 480 | -4.12 | -6,-26,-18 |
| Right Locus Coeruleus | | |  | -3.90 | -4,-36,-16 |
|  |  | | | -3.66 | -10,-24,-24 |
|  |  | | | -4.26 | 8,-28,-12 |
|  |  | | | -4.20 | 8,-24,-14 |
|  |  | | | -3.66 | 6,-38,-20 |
| Ventral Tegmental Area | | | 32 | -2.02 | 0, -16, -10 |
|  | | | | | -2, -20, -12 |
| Right Cingulate Gyrus/Medial Frontal Gyrus | | 24 | 29,704 | -5.03 | 16,-20,40 |
| Right Superior Frontal Gyrus | | 6 |  | -3.40 | 20,-4,62 |
| Left Medial Frontal Gyrus | | 6 |  | -3.82 | -12,-6,64 |
| Left Middle Frontal Gyrus | | 6 |  | -3.75 | -24,-4,38 |
| Left Cingulate Gyrus | | 24 |  | -4.24 | -16,-8,36 |
| Right Cingulate Gyrus | | 24 |  | -4.18 | 10,-6,30 |
| Right Inferior Parietal Lobule | | 40 |  | -4.02 | 38,-24,36 |
| Right Posterior Cingulate Gyrus | | 31 |  | -3.85 | 16,-30,30 |
| Right Superior Parietal Lobule | | 7 |  | -4.38 | 20,-44,42 |
| Left Hippocampus | |  |  | -4.46 | -12,-38,8 |
| Left Putamen | |  |  | -3.77 | -32,-6,6 |
| Right Middle Frontal Gyrus | | 6 | 5,128 | -4.09 | 24,-6,42 |
| Right Cingulate Gyrus | | 24 |  | -3.95 | 18,-10,46 |
|  | |  |  | -3.87 | 20,-6,46 |
| Right Superior Frontal Gyrus | | 6 |  | -3.77 | 22,-10,56 |
|  | |  |  | -3.74 | 20,-8,60 |
| Right Inferior Frontal Gyrus | | 47 | 8,408 | -3.32 | 40,38,-8 |
| Right Inferior Frontal Gyrus/ Gyrus Rectus | | 47/10 |  | -3.29 | 30,32,-10 |
| Right Putamen | |  |  | -3.27 | 32,-2,8 |
| Left Inferior Parietal Lobule | | 40 | 1,400 | -3.53 | -44,-26,30 |
| Left Posterior Cingulate Gyrus | | 31 | 5,168 | -3.70 | -20,-26,44 |
| Left Paracentral Lobule | | 5 |  | -3.64 | -16,-30,52 |
| Left Superior Temporal Gyrus/Inferior Parietal Lobule | | 7 |  | -3.36 | -20,-40,42 |
| Right Middle Temporal Gyrus | | 21 | 968 | -3.56 | 56,-30,-16 |
| Left Parahippocampal Gyrus | | 20/36 | 1,064 | -4.11 | -28,-32,-14 |
| Left Inferior Temporal Gyrus | | 20 |  | -3.71 | -38,-30,-14 |
| Left Cerebellar Vermis | |  | 7,600 | -3.49 | -2,-52,-22 |
| **Control-related Drug Effect: Greater Activation in SZ Group vs HC** | | | | | |
| (None) | |  |  |  |  |
