# Supplementary figures and images for: Altered brainstem responses to modafinil in schizophrenia: implications for adjunctive treatment of cognition
Source: Transl Psychiatry. 2018 Mar 6;8:58. doi: 10.1038/s41398-018-0104-z (PMC5838154; doi:10.1038/s41398-018-0104-z)

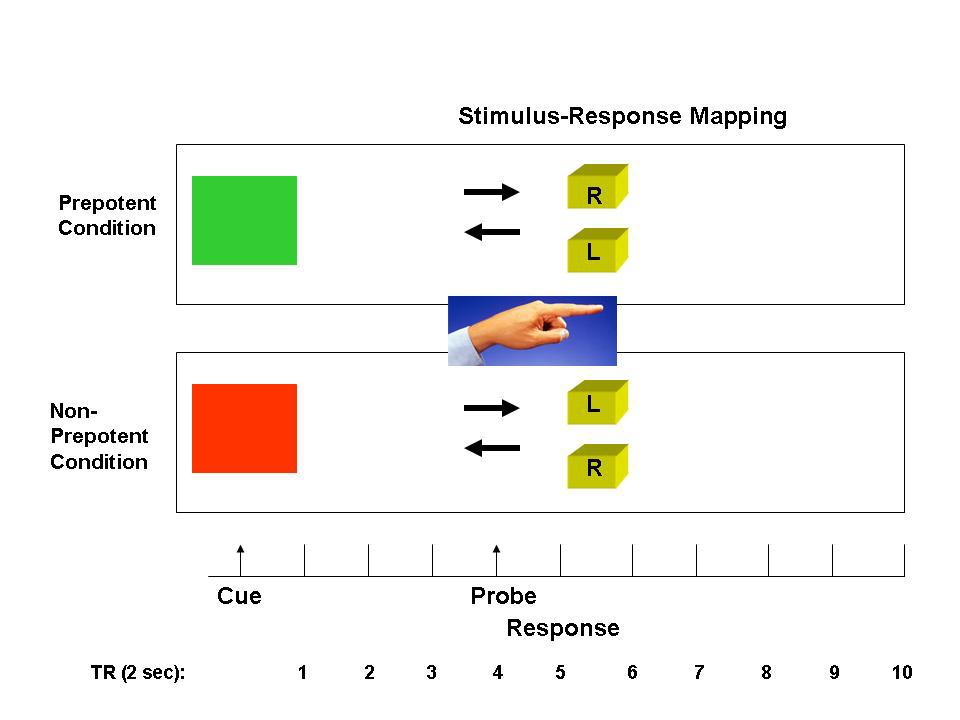

Supplement: Supplementary file 2 — Supplemental Figure 1 [file 41398_2018_104_MOESM2_ESM.tif]

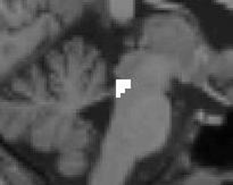

Supplement: Supplementary file 3 — Supplemental Figure 2 [file 41398_2018_104_MOESM3_ESM.tif]

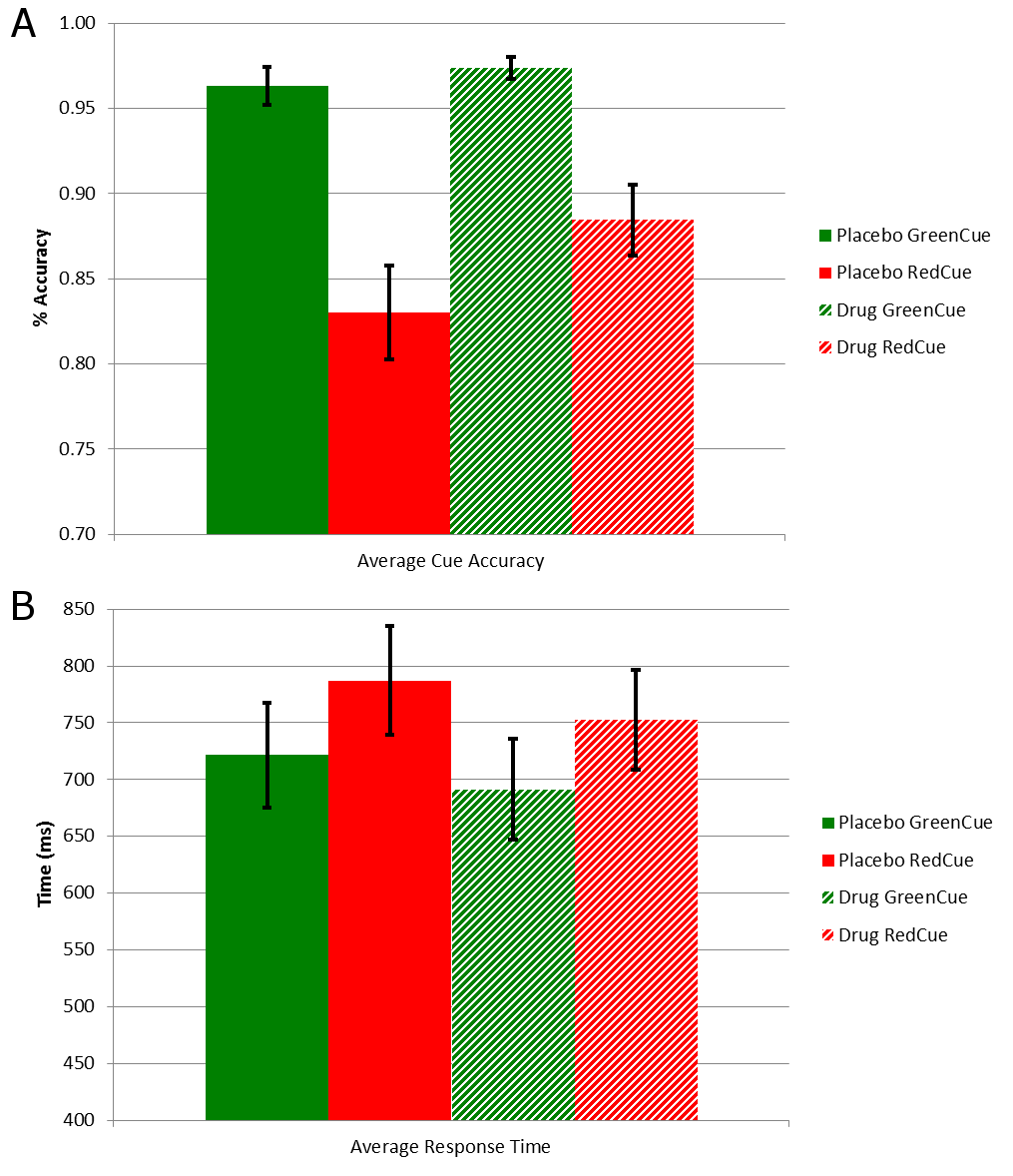

Supplement: Supplementary file 4 — Supplemental Figure 3 [file 41398_2018_104_MOESM4_ESM.tif]
